# Incidence, worsening and risk factors of daytime sleepiness in a population-based 5-year longitudinal study

**DOI:** 10.1038/s41598-017-01547-0

**Published:** 2017-05-02

**Authors:** I. Jaussent, C. M. Morin, H. Ivers, Y. Dauvilliers

**Affiliations:** 1 Inserm, U1061, F-34000 Montpellier, France; 2 Université Montpellier, F-34000 Montpellier, France; 3 Department of Psychology, Université Laval, Quebec City, Quebec Canada; 4 CHU Montpellier, Service de Neurologie, Unité des Troubles du Sommeil, Hôpital Gui-de-Chauliac, Montpellier, France

## Abstract

Excessive daytime sleepiness (EDS) is highly prevalent in the general population; however little is known about its evolution and predictors. Our objectives were to document its natural history, provide estimates of its prevalence, incidence and persistence rates, and to identify predictors of increased daytime sleepiness (DS) in a longitudinal community study of 2157 adults over 5 years. Participants completed postal assessment at baseline and at each yearly follow-up. DS was evaluated by the Epworth Sleepiness scale (ESS). At baseline, 33% reported EDS (ESS > 10) with 33% of them reported persistent EDS. Of those without EDS at baseline, 28% developed incident EDS (15% were persistent) and 31% increased DS (augmentation ≥4-points between two consecutive evaluations). Younger age and depression were independent predictors of incident EDS and DS increase while lower coffee consumption, smoking, insomnia, tiredness and chronic pain were associated with incident EDS, and living alone with DS increase only. Persistent *vs* transient EDS or DS showed association with poor general health including metabolic diseases. Thus, sleepiness fluctuated over time and it was predicted by common lifestyle and psychological factors potentially modifiable. However, persistent sleepiness was associated with chronic medical diseases thus highlighting a homogeneous group at risk requiring a dedicated management.

## Introduction

Excessive daytime sleepiness (EDS) is characterized by a difficulty to stay awake and alert during the major waking episodes of the day, with sleep occurring unintentionally or at inappropriate times of the wake period^1^. EDS is often associated with a wide range of illnesses including metabolic, cardiovascular, neurological, psychiatric diseases^2–5^ but also with voluntary behaviors reflecting poor sleep and sleep debt, leading to disability and increased risk of mortality^6^. EDS is also commonly associated with social and economic consequences thus constituting a significant public health problem. The prevalence of EDS in the general population varies from 9 to 28%^5, 7–10^. This wide range can be explained by differences according to demographic and cultural/geographical factors (e.g. higher prevalence in young^11^ and older adults^3^, higher prevalence in northern countries^12^) but also inconsistencies in study design, sample size, EDS assessment and definition^13^. Survey studies used either single questions to evaluate excessive sleepiness, sleep propensity during wakefulness and unrefreshing sleep, self-report questionnaires to measure daytime sleepiness (DS)^13^, or a more complex structured interview using criteria proposed for the DSM-5^10^. The most commonly used instrument in clinical and research settings is the Epworth Sleepiness Scale (ESS), a standardized self-report questionnaire^14^. A cut-off greater than 10 was used to indicate a clinically significant EDS in clinical and epidemiological studies with a prevalence ranging from 8.5% to 22.2% in the general population^5, 8, 11, 15, 16^. In addition to this cut-off, a change of four points or more on ESS scores between any two evaluations was considered clinically relevant and was frequently used as a primary endpoint in pharmacological studies on central hypersomnias^17, 18^.

To our knowledge, factors predicting EDS are not well understood and those triggering DS increase remain unknown. Longitudinal studies of factors associated with EDS onset are scarce and give inconsistent results. Using single questions to evaluate EDS with Likert-type scales or dichotomized responses, only three studies reported that early morning awakening and anxiety increase the risk of EDS in young adults^19^, insomnia, anxiety/depression and smoking predict incident EDS in women^20^, and obesity and depression are associated with the occurrence of EDS in middle-aged adults^21^. However, none of these cohorts used the ESS scale to define EDS and despite long follow-ups (10 years or more), they neither fully took into account changes in covariates nor the fluctuation of sleepiness course over time.

The objectives of the present study were to document the natural history of EDS and provide estimates of prevalence, incidence, and persistence rates of EDS using the ESS, and to identify predictors of increased DS using time-dependent covariates over a 5-year follow-up in a longitudinal community study of adults.

## Results

### Subject characteristics

**Figure 1:**
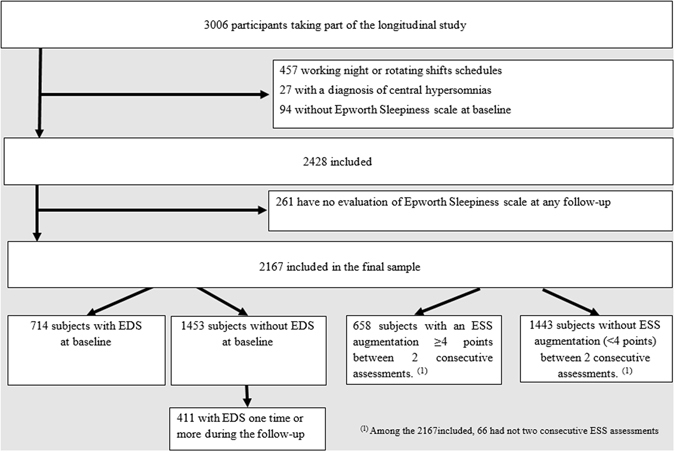
Flow chart diagram.

The final sample included 2167 participants with a median baseline age of 51 years (range, 18–89) of whom 64.1% were women. As detailed in the flow-chart diagram (Fig. 1), these subjects were free of central hypersomnia disorders, day workers, and with ESS completed at baseline and at least at one of the five annual follow-up evaluations. Participants excluded from the study (N = 261) had a significantly lower education level, were younger, current smoker and more depressed. No significant differences were found for sleep duration, number of naps, insomnia, EDS, or associated chronic diseases.

Regarding other baseline sleep characteristics, 13.1% slept less than 6 hours per night, 13.7% took three naps or more during the week, and 16.1% had a moderate to severe level of insomnia (ISI > 14). Only 14.1% took hypnotics (50.2% BZD, 19.6% BZD-like compounds), 12.6% antidepressants, and 29.4% OTC medication (23.8% antihistaminics and 5.6% melatonin).

### Description of the natural history of EDS

The Cronbach’s coefficient alpha for the 8 items of ESS scale was around 0.80–0.81 at baseline and at each year-follow-up. The inter-rater agreement measures between two assessment points were good ranging from 0.64 95% CI = [0.62–0.67] to 0.79 95% CI = [0.77–0.81].

**Figure 2:**
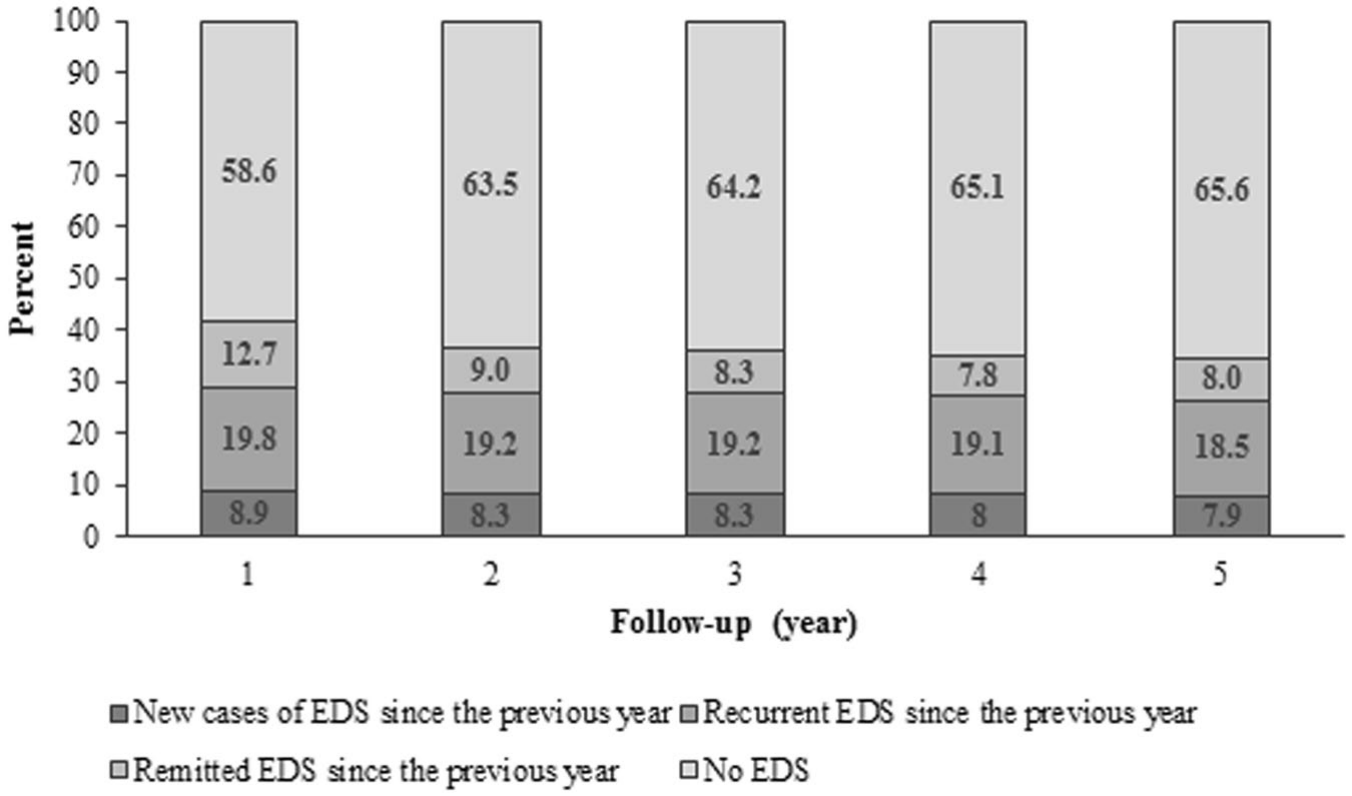
Prevalences of new, recurrent and remitted cases of excessive daytime sleepiness (EDS) during the follow-up.

At baseline, 32.9% (95% CI = [31.0–34.9]) (n = 714) of participants reported EDS (ESS > 10), of whom 25.4% were severely sleepy (ESS ≥ 16). During the follow-up visits, prevalences of EDS were slightly lower ranging from 28.7 (95% CI = [26.8–30.8]) to 26.4% (95% CI = [24.3–28.5]). However, trajectories of EDS vary considerably with approximately 8% of new cases each year, between 8 and 13% of remitted cases since the previous year and approximately 19% of recurrent cases since the previous year (Fig. 2). Regarding the persistence of EDS during the follow-up, 32.6% (n = 233) reported a persistent EDS at baseline and at all 5 subsequent assessments, 25 developed EDS after 1-year of follow-up with a persistence at all subsequent assessments, 14 after two years, 13 after three years and 11 after four years.

### Determinants of EDS at baseline

Participants with EDS were younger, slept less than eight hours per night, had a higher frequency of naps, and took hypnotics less frequently (Table 1). They were more likely to complain insomnia, fatigue, depressive and anxiety symptoms, were obese and less physically active, and reported higher rates of diagnoses of sleep apnea, chronic pain, neurological or other diseases (p ≤ 0.02 for all comparisons) (Table 1). To identify which determinants were independently associated with EDS, gender and other characteristics associated with EDS at p < 0.15 were introduced into a multivariate model. Younger age, shorter sleep duration, higher frequency of naps, fatigue, levels of trait-anxiety, sleep apnea and the absence of hypnotic consumption were associated with EDS, but not insomnia, depressive symptoms or chronic diseases (Table 1).

**Table 1:** Bivariate and common analyses of the association between potential determinants and excessive daytime sleepiness (Epworth Sleepiness Scale (ESS) total score >10) at baseline.

*Variable*	*ESS total score*							
	≤*10 N *= *1453*		>*10 N *= *714*		*Bivariate analysis*		*Multivariate analysis* ^*(1)*^	
	*n*	*%*	*n*	*%*	*OR [95% CI]*	*P-value*	*OR [95% CI]*	*P-value*
Gender (Women)	942	64.83	447	62.61	0.91 [0.75;1.09]	0.31	0.94 [0.75;1.18]	0.59
Age, in years (tertiles)								
≤44	485	33.38	256	35.85	1.31 [1.05;1.64]	0.02	1.38 [1.04;1.81]	0.04
]44–58]	476	32.76	260	36.41	1.36 [1.09;1.70]		1.36 [1.05;1.77]	
>58	492	33.86	198	27.73	1		1	
High educational level (yes)	509	35.03	244	34.17	0.96 [0.80;1.16]	0.69		
Living as a couple (yes)	860	59.19	441	61.85	1.12 [0.93;1.34]	0.23		
Smoking status								
No smoker	1140	78.62	563	79.18	1	0.6		
Current smoker	219	15.1	111	15.61	1.03 [0.80;1.32]			
Occasional smoker	91	6.28	37	5.2	0.82 [0.55;1.22]			
Coffee consumption								
No	257	17.85	117	16.62	1	0.61		
One-Two cups per day	676	46.94	325	46.16	1.06 [0.82;1.36]			
Three or more cups per day	507	35.21	262	37.22	1.14 [0.87;1.48]			
Alcohol consumption								
No	1099	77.01	524	75.94	1	0.63		
One drink per day	196	13.74	93	13.48	1.00 [0.76;1.30]			
Two or more drinks per day	132	9.25	73	10.58	1.16 [0.86;1.57]			
Sleep duration, *in hour*								
≥8:00	403	28.36	144	20.66	1	<0.0001	1	0.04
[6:00–7:59]	866	60.94	429	61.55	1.39 [1.11;1.73]		1.31 [1.02;1.69]	
<6:00	152	10.7	124	17.79	2.28 [1.68;3.09]		1.54 [1.06;2.23]	
Naps during the last month								
No	624	43.18	179	25.14	1	<0.0001	1	<0.0001
<1 time/week	442	30.59	196	27.53	1.55 [1.22;1.96]		1.37 [1.06;1.77]	
1–2 times/week	224	15.5	196	27.53	3.05 [2.37;3.93]		2.50 [1.88;3.31]	
≥3 times/week	155	10.73	141	19.8	3.17 [2.39;4.21]		2.72 [1.97;3.76]	
Hypnotics intake during the last month (yes)	226	15.67	76	10.83	0.65 [0.50;0.86]	0.003	0.46 [0.33;0.64]	<0.0001
Insomnia severity index								
<8	816	56.67	273	38.67	1	<0.0001	1	0.31
8–14	431	29.93	280	39.66	1.94 [1.58;2.38]		1.28 [0.99;1.65]	
15–21	175	12.15	136	19.26	2.32 [1.79;3.02]		1.18 [0.81;1.72]	
≥22	18	1.25	17	2.41	2.82 [1.43;5.55]		1.21 [0.48;3.06]	
Insomnia diagnosis (yes)	99	6.88	49	6.92	1.01 [0.71;1.44]	0.97		
Sleep apnea diagnosis (yes)	40	2.78	40	5.65	2.09 [1.34;3.28]	0.001	1.77 [1.02;3.06]	0.04
Restless legs syndrome (yes)	34	2.36	24	3.39	1.45 [0.85;2.46]	0.17		
General fatigue								
≤9	693	48.26	188	26.7	1	<0.0001	1	<0.0001
10–12	403	28.06	230	32.67	2.10 [1.67;2.64]		1.89 [1.43;2.48]	
>12	340	23.68	286	40.63	3.10 [2.48;3.88]		2.20 [1.59;3.04]	
Beck Depression Inventory score								
≤13	1217	84.93	514	73.01	1	<0.0001	1	0.18
14–19	109	7.61	95	13.49	2.06 [1.54;2.77]		1.30 [0.92;1.85]	
20–28	77	5.37	62	8.81	1.91 [1.34;2.71]		1.39 [0.91;2.12]	
29–63	30	2.09	33	4.69	2.60 [1.57;4.31]		1.61 [0.85;3.08]	
Spielberger’s State Anxiety								
<46	514	35.62	299	42.41	1	0.009	1	0.31
46–49	440	30.49	187	26.52	0.73 [0.58;0.91]		0.82 [0.63;1.06]	
≥50	489	33.89	219	31.06	0.77 [0.62;0.95]		0.91 [0.70;1.19]	
Spielberger’s Trait Anxiety								
<45	637	44.17	293	41.44	1	0.13	1	0.0002
45–47	304	21.08	137	19.38	0.98 [0.77;1.25]		1.24 [0.94;1.64]	
≥48	501	34.74	277	39.18	1.20 [0.98;1.47]		1.67 [1.31;2.13]	
Physical activity								
≥4 times per week	501	34.67	186	26.31	1	<0.0001	1	0.13
[1–3] times per week	654	45.26	331	46.82	1.36 [1.10;1.69]		1.15 [0.90;1.46]	
Less than 1 per week/never	290	20.07	190	26.87	1.76 [1.38;2.26]		1.35 [1.01;1.81]	
Body Mass index, in kg/m^2^								
<25	681	47.13	298	42.27	1	0.01	1	0.57
[25–30]	491	33.98	236	33.48	1.10 [0.89;1.35]		1.13 [0.89;1.43]	
≥30	273	18.89	171	24.26	1.43 [1.13;1.81]		1.11 [0.84;1.48]	
Chronic pain (yes)	343	23.64	208	29.13	1.33 [1.09;1.62]	0.006	0.91 [0.71;1.16]	0.44
Cardio-vascular disease (yes)	79	5.44	41	5.74	1.06 [0.72;1.56]	0.77		
Hypertension (yes)	265	18.3	136	19.24	1.06 [0.85;1.34]	0.6		
Endocrine and metabolic disease (yes)	211	14.53	114	15.97	1.12 [0.87;1.43]	0.38		
Diabetes (yes)	94	6.49	45	6.31	0.97 [0.67;1.40]	0.88		
Neurological disease (yes)	125	8.61	88	12.32	1.49 [1.12;1.99]	0.007	1.08 [0.76;1.54]	0.68
Other diseases (yes)	662	45.78	374	52.53	1.31 [1.09;1.57]	0.003	1.06 [0.86;1.32]	0.58

^(1)^For the multivariate analysis, only gender and variables associated at p < 0.15 in univariate analysis were entered into the model.

To examine whether determinants could be differentially associated with EDS severity, subjects were categorized into three groups: no EDS (ESS ≤ 10, n = 1453), EDS (ESS between 11 and 15, n = 533) and severe EDS (ESS ≥ 16, n = 181). Compared to those without EDS, those with EDS or severe EDS were independently more tired, trait-anxious, had frequent naps and consumed no hypnotic whereas those with non-severe EDS only were younger and reported more short sleep duration, and those with severe EDS only had more sleep apnea.

### Predictors of EDS over 5-year follow-up

Among the 1453 subjects without EDS at baseline, 411 (28.3%) met criteria for incident EDS at least in one of the subsequent follow-ups. The median occurrence of the first EDS event was 2 years [range of 0.40–5.71].

In bivariate Cox models including time-dependent variables, the risk of incident EDS was higher in subjects living alone, smoking occasionally, consuming less coffee, sleeping less than eight hours, taking frequently naps, practicing physical activities less than 4 times per week, being insomniac, tired and depressed, less anxious, and with chronic diseases (restless legs syndrome, chronic pain, endocrine, metabolic neurological and other diseases) (Table 2). BMI category or a change in BMI categories was not significantly associated with incident EDS. In a Cox multivariate model including gender, ESS baseline and characteristics associated with a p < 0.15, being younger (borderline significant), consuming less coffee, smoking occasionally, being insomniac, depressed, with fatigue and having chronic pain were independent predictors of the incidence of EDS (Table 3). To test whether a potential multicollinearity of fatigue, depression symptoms and insomnia, several multivariate models were rerun including all covariates associated at p < 0.15 1) without the covariate fatigue 2) without the depression symptoms 3) without insomnia severity. The results of associations between potential predictors and incidence of EDS during the 5-year follow-up remained unchanged.

**Table 2:** Predictors of incident excessive daytime sleepiness (EDS) and daytime sleepiness (DS) increased over of 5-year follow-up.

*Variable*	*Incidence of EDS*						≥*4-point increase of DS*					
	*No*		*Yes*		*HR [95% CI]*	*P-value*	*No*		*Yes*		*HR [95% CI]*	*P-value*
	*N* = *1042*		*N* = *411*				*N* = *1443*		*N* =* 658*			
	*n*	*%*	*n*	*%*			*n*	*%*	*n*	*%*		
Gender (women)	678	65.07	264	64.23	0.86 [0.78;1.17]	0.67	915	63.41	435	66.11	1.13 [0.96;1.33]	0.14
Age, in years (tertiles)												
≤44	340	32.63	145	35.28	1.24 [0.98;1.58]	0.12	488	33.82	219	33.28	1.08 [0.89;1.31]	0.64
[44–58]	331	31.77	145	35.28	1.26 [0.99;1.60]		483	33.47	235	35.71	1.08 [0.90;1.31]	
>58	371	35.6	121	29.44	1		472	32.71	204	31	1	
High educational level (yes)	373	35.8	136	33.09	0.86 [0.70;1.05]	0.14	517	35.83	214	32.52	0.83 [0.70;0.97]	0.02
Living as a couple (yes)	630	60.46	230	55.96	0.81 [0.67;0.99]	0.04	886	61.44	376	57.14	0.83 [0.72;0.97]	0.02
Smoking status												
No smoker	853	81.94	323	78.78	1	0.03	1189	82.51	524	79.76	1	0.16
Current smoker	137	13.16	52	12.68	1.00 [0.74;1.33]		189	13.12	100	15.22	1.23 [0.99;1.53]	
Occasional smoker	51	4.9	35	8.54	1.60 [1.13;2.27]		63	4.37	33	5.02	1.06 [0.74;1.50]	
Coffee consumption												
No	141	13.54	98	23.9	1	0.0003	210	14.57	131	19.94	1	0.15
One-Two cups per day	533	51.2	175	42.68	0.61 [0.47;0.78]		713	49.48	309	47.03	0.84 [0.68;1.03]	
Three or more cups per day	367	35.25	137	33.41	0.67 [0.52;0.87]		518	35.95	217	33.03	0.82 [0.66;1.02]	
Alcohol consumption												
No	801	77.17	327	79.95	1	0.21	1085	75.45	515	78.87	1	0.08
One drink per day	128	12.33	50	12.22	0.87 [0.64;1.17]		185	12.87	83	12.71	0.88 [0.70;1.11]	
Two or more drinks per day	109	10.5	32	7.82	0.74 [0.52;1.07]		168	11.68	55	8.42	0.75 [0.57;0.99]	
Sleep duration, *in hour*												
≥8	369	35.51	97	23.83	1	<0.0001	462	32.22	170	26.11	1	0.02
[6–8]	565	54.38	250	61.43	1.47 [1.17;1.86]		798	55.65	390	59.91	1.21 [1.01;1.44]	
<6	105	10.11	60	14.74	2.05 [1.49;2.83]		174	12.13	91	13.98	1.43 [1.11;1.84]	
Naps during the last month												
No	513	49.33	133	32.6	1	<0.0001	616	42.78	247	37.71	1	0.32
<1 time/week	291	27.98	133	32.6	1.48 [1.16;1.88]		390	27.08	183	27.94	0.98 [0.81;1.19]	
1–2 times/week	149	14.33	81	19.85	1.81 [1.37;2.39]		261	18.13	137	20.92	1.16 [0.94;1.43]	
≥3 times/week	87	8.37	61	14.95	2.38 [1.76;3.22]		173	12.01	88	13.44	1.15 [0.90;1.47]	
Hypnotics intake during the last month (yes)	63	6.05	38	9.31	1.24 [0.88;1.73]	0.22	86	5.97	53	8.08	1.25 [0.95;1.66]	0.12
Insomnia severity index												
<8	683	65.55	198	48.41	1	<0.0001	876	60.71	332	50.69	1	<0.0001
8–14	255	24.47	144	35.21	1.83 [1.48;2.27]		404	28	220	33.59	1.37 [1.15;1.62]	
15–21	98	9.4	56	13.69	1.86 [1.38;2.50]		150	10.4	84	12.82	1.40 [1.10;1.78]	
≥22	6	0.58	11	2.69	3.45 [1.88;6.33]		13	0.9	19	2.9	3.17 [2.00;5.04]	
Insomnia diagnosis (yes)	142	13.75	48	11.82	1.16 [0.88;1.57]	0.34	187	13.1	94	14.46	1.39 [1.12;1.73]	0.003
Sleep apnea diagnosis (yes)	45	4.36	20	4.94	1.48 [0.95;2.32]	0.09	83	5.81	37	5.71	1.24 [0.89;1.72]	0.21
Restless legs syndrome (yes)	48	4.66	22	5.43	1.64 [1.07;2.53]	0.02	82	5.75	32	4.94	1.19 [0.83;1.70]	0.34
General fatigue												
≤9	585	56.3	145	35.37	1	<0.0001	720	50.07	252	38.36	1	<0.0001
10–12	239	23	143	34.88	2.08 [1.65;2.62]		367	25.52	212	32.27	1.47 [1.23;1.77]	
>12	215	20.69	122	29.76	2.10 [1.65;2.67]		351	24.41	193	29.38	1.46 [1.21;1.77]	
Beck Depression Inventory score												
≤13	929	89.16	320	78.43	1	<0.0001	1243	86.2	505	77.22	1	<0.0001
14–19	67	6.43	42	10.29	1.77 [1.29;2.45]		106	7.35	69	10.55	1.46 [1.13;1.88]	
20–28	33	3.17	30	7.35	2.23 [1.53;3.24]		72	4.99	51	7.8	1.97 [1.48;2.63]	
29–63	13	1.25	16	3.92	2.57 [1.55;4.24]		21	1.46	29	4.43	2.29 [1.58;3.34]	
Spielberger’s State Anxiety												
<46	358	34.36	172	42.16	1	0.01	524	36.36	255	38.93	1	0.5
46–49	302	28.98	113	27.7	0.80 [0.63;1.01]		416	28.87	175	26.72	0.90 [0.74;1.09]	
≥50	382	36.66	123	30.15	0.71 [0.56;0.89]		501	34.77	225	34.35	0.92 [0.77;1.11]	
Spielberger’s Trait Anxiety												
<45	473	45.44	184	44.99	1	0.52	659	45.8	294	44.82	1	0.68
45–47	261	25.07	86	21.03	0.92 [0.71;1.19]		345	23.97	139	21.19	0.95 [0.77;1.16]	
≥48	307	29.49	139	33.99	1.08 [0.86;1.34]		435	30.23	223	33.99	1.04 [0.87;1.24]	
Physical activity												
≥4 times per week	344	33.08	117	28.47	1	0.03	464	32.22	189	28.72	1	0.04
[1–3] times per week	471	45.29	192	46.72	1.27 [1.01;1.59]		657	45.63	304	46.2	1.18 [0.99;1.42]	
Less than 1 per week/never	225	21.63	102	24.82	1.42 [1.09;1.85]		319	22.15	165	25.08	1.30 [1.06;1.61]	
Body Mass index, in kg/m^2^												
<25	479	45.97	173	42.3	1	0.2	626	43.41	267	40.7	1	0.002
[25–30]	366	35.12	145	35.45	1.12 [0.89;1.39]		544	37.73	220	33.54	1.02 [0.85;1.22]	
≥30	197	18.91	91	22.25	1.26 [0.98;1.62]		272	18.86	169	25.76	1.38 [1.14;1.67]	
Change of BMI classes ^(1)^												
Loss of BMI class	74	7.32	30	7.67	1.25 [0.86;1.82]	0.26	98	6.82	44	6.91	1.07 [0.78;1.45]	0.76
No change	864	85.46	324	82.86	1		1234	85.87	539	84.62	1	
Gain of BMI class	73	7.22	37	9.46	1.25 [0.89;1.75]		105	7.31	54	8.48	1.10 [0.83;1.46]	
Chronic pain (yes)	229	21.98	126	30.66	1.67 [1.36;2.06]	<0.0001	338	23.42	188	28.57	1.27 [1.07;1.50]	0.006
Cardio-vascular disease (yes)	98	9.51	39	9.68	1.39 [1.00;1.93]	0.05	129	9.01	68	10.56	1.44 [1.12;1.85]	0.005
Hypertension (yes)	278	27.2	97	24.43	1.07 [0.85;1.35]	0.55	381	26.89	179	28.14	1.19 [1.00;1.42]	0.05
Endocrine and metabolic disease (yes)	243	23.52	97	23.95	1.37 [1.09;1.73]	0.007	346	24.11	164	25.35	1.32 [1.10;1.57]	0.003
Diabetes (yes)	97	9.41	39	9.68	1.39 [1.00;1.94]	0.05	131	9.15	66	10.26	1.41 [1.09;1.82]	0.008
Neurological disease (yes)	126	12.21	70	17.33	1.84 [1.43;2.39]	<0.0001	208	14.53	115	17.86	1.54 [1.26;1.88]	<0.0001
Other diseases (yes)	462	44.34	205	49.88	1.37 [1.13;1.66]	0.002	657	45.53	328	49.85	1.33 [1.14;1.55]	0.0003

^(1)^Change between the two last evaluations before the 4-point increase.

**Table 3:** Common analyses of the associations between potential predictors and incidences of excessive daytime sleepiness (EDS) and of a 4-point increase of daytime sleepiness (DS) during the 5-year follow-up.

*Variable*	*Incidence of EDS*		≥*4-point increase of DS*	
	*Yes vs No*		*Yes vs No*	
	*HR [95% CI]*	*P-value* ^*(0)*^	*HR [95% CI]*	*P-value* ^*(0)*^
Gender (women)	1.00 [0.80;1.25]	0.99	1.05 [0.88;1.26]	0.6
Age, in years (tertiles)				
≤44	1.40 [1.04;1.89]	0.08	1.35 [1.06;1.70]	0.05
[44–58]	1.26 [0.96;1.66]		1.18 [0.95;1.45]	
>58	1		1	
High educational level (yes)	0.89 [0.71;1.12]	0.32	0.89 [0.75;1.06]	0.19
Living as a couple (yes)	0.88 [0.72;1.09]	0.25	0.83 [0.71;0.98]	0.02
Smoking status				
No smoker	1	0.02		
Current smoker	0.89 [0.64;1.22]			
Occasional smoker	1.59 [1.11;2.28]			
Coffee consumption				
No	1	0.0007		
One-Two cups per day	0.62 [0.48;0.81]			
Three or more cups per day	0.63 [0.48;0.83]			
Alcohol consumption				
No			1	0.32
One drink per day			1.00 [0.79;1.28]	
Two or more drinks per day			0.80 [0.59;1.08]	
Sleep duration, *in hour*				
≥8	1	0.08	1	0.2
[6–8]	1.28 [0.99;1.64]		1.19 [0.98;1.44]	
<6	1.46 [1.01;2.10]		1.19 [0.88;1.59]	
Naps during the last month				
No	1	0.23		
<1 time/week	0.99 [0.77;1.27]			
1–2 times/week	1.16 [0.87;1.56]			
≥3 times/week	1.35 [0.97;1.89]			
Hypnotics intake during the last month (yes)			1.04 [0.76;1.43]	0.79
Insomnia severity index				
<8	1	0.006	1	0.24
8–14	1.27 [0.98;1.65]		1.12 [0.91;1.37]	
15–21	0.96 [0.65;1.44]		0.88 [0.64;1.22]	
≥22	3.07 [1.42;6.64]		1.31 [0.70;2.45]	
Sleep apnea diagnosis (yes)	1.03 [0.62;1.69]	0.91		
Restless legs syndrome (yes)	1.14 [0.72;1.81]	0.57		
General fatigue				
≤9	1	0.005	1	0.06
10–12	1.49 [1.14;1.95]		1.16 [0.94;1.43]	
>12	1.09 [0.77;1.53]		0.89 [0.69;1.16]	
Beck Depression Inventory score				
≤13	1	0.02	1	0.0004
14–19	1.30 [0.91;1.86]		1.33 [1.00;1.76]	
20–28	1.62 [1.08;2.44]		1.82 [1.31;2.52]	
29–63	2.11 [1.15;3.86]		1.97 [1.25;3.12]	
Spielberger’s State Anxiety				
<46	1	0.9		
46–49	1.04 [0.81;1.34]			
≥50	1.06 [0.82;1.36]			
Physical activity				
≥4 times per week	1	0.75	1	0.69
[1–3] times per week	1.06 [0.83;1.35]		1.05 [0.87;1.28]	
Less than 1 per week/never	1.12 [0.84;1.50]		1.11 [0.88;1.39]	
Body Mass index, in kg/m^2^				
<25			1	0.34
[25–30]			1.12 [0.93;1.35]	
≥30			1.16 [0.93;1.45]	
Chronic pain (yes)	1.31 [1.03;1.66]	0.03	1.07 [0.88;1.29]	0.5
Cardio-vascular disease (yes)	1.05 [0.71;1.56]	0.81	1.25 [0.93;1.66]	0.14
Hypertension (yes)			1.06 [0.86;1.30]	0.58
Endocrine and metabolic disease (yes)	1.21 [0.94;1.58]	0.15	1.20 [0.98;1.47]	0.08
Diabetes (yes)	1.40 [0.97;2.02]	0.07	1.08 [0.81;1.46]	0.59
Neurological disease (yes)	1.31 [0.97;1.77]	0.08	1.09 [0.86;1.36]	0.49
Other diseases (yes)	1.17 [0.93;1.47]	0.17	1.16 [0.98;1.39]	0.09

^(0)^Only baseline ESS score, age, gender and variables associated at p < 0.15 in univariate analysis were entered into the model.

Among the participants with incident EDS, only 15.3% (n = 63) reported a persistent EDS in all subsequent assessments. Compared to participants with transient EDS, participants with persistent EDS reported a higher frequency of diabetes (HR = 3.23 95% CI = [1.51;6.91], p = 0.003) in bivariate analysis and this result remained unchanged after adjustment for gender, age and ESS baseline (HR = 3.10 95% CI = [1.40;6.85], p = 0.005).

### Predictors of increased DS over 5-year follow-up

Over the 5-year follow-up, 658 (31.3%) participants had increased DS (≥4 points on ESS) with a median delay between baseline and follow-up augmentation of 2.16 years [range of 0.25–5.59].

In bivariate Cox models including time-dependent variables, participants with increased DS were significantly more likely to have a low educational level, live alone, sleep less than eight hours per night, practice physical activities less than 4 hours per week and reported more severe insomnia, fatigue, and depressive symptoms. They were more obese and reported more chronic diseases (chronic pain, diabetes, cardiovascular, endocrine and metabolic, neurological and other diseases) (Table 2). In a multivariate model, participants with increased DS were independently younger, lived alone and more depressed (Table 3). Among participants with increased DS, 35.6% reported a persistent DS through the study. In bivariate analysis, compared to participants with transient DS increase, those with persistent increase DS had more diabetes (HR = 1.61 95% CI = [1.05;2.45], p = 0.03), endocrine and metabolic diseases (HR = 1.41 95% CI = [1.06;1.92], p = 0.03) and other diseases (HR = 1.39 95% CI = [1.05;1.84], p = 0.02). In multivariate analysis, including these three diseases, age, gender and ESS baseline, only other diseases were independently associated with a persistent increase DS (HR = 1.36 95% CI = [1.01;1.83], p = 0.04).

## Discussion

In this study of community-dwelling middle aged adults, 33% of the participants met the criteria for self-reported EDS at baseline (ESS > 10) (of whom 25% had severe EDS), with 33% of them reporting persistent EDS over 5 years. During the follow-up, 28% had incident EDS and 31% had increased DS (≥4-point on ESS). However, the course of EDS fluctuates over time with only 15% having persistent EDS and 36% persistent DS. We found that younger age and depressive symptoms were independent predictors of incident EDS and DS increase while lower coffee consumption, smoking, insomnia, tiredness and chronic pain were associated with incident EDS, and living alone with DS increase only. Only poor general health with somatic diseases, especially metabolic diseases, was associated with persistent EDS or DS increased.

We evaluated DS using the ESS which is based more on observable behaviors than subjective experience, reflecting real-life experience, with a good correlation found between the scores obtained by patients and their partners. The score is reliable and reproducible with good test-retest reliability (r = 0.82) between two administrations of the ESS over a time period of 5-months^22^. Prevalence rates for EDS were in the same range at baseline and at each point of the follow-up. These estimations were, however, higher than previous studies using the same questionnaire, ranging from 8.5 to 22.2%^5, 8, 11, 15, 16^. Reasons for such estimations may be due to the administration mode of the ESS scale, being here self-reported and completed via a postal mailing thus without clear face-to-face information on how to answer it. It has been reported higher score when the questionnaire was only self-administrated^23^. Consequently, we cannot excluded a certain degree of misclassification, with some overlapping with fatigue.

In our study, no gender difference was found while EDS slightly decreased with age as previously reported^8, 15^. In contrast, shorter sleep duration, higher frequency of naps, fatigue, higher levels of trait-anxiety and the absence of hypnotic consumption were associated with EDS. Only sleep apnea was related to severe EDS (ESS > 16).

We also found a high frequency of incident EDS (28%) over the 5-year period compared to previous studies^8, 15^. However, among participants who developed EDS in our study only 15% were persistent which suggests instability, with frequent waxing and waning in the natural course of EDS in the general population. This finding could not be assessed in previous studies, as no data on DS were available between baseline and endpoint follow-up^8, 15^. Low persistent EDS rate was, however, in agreement with a recent study measuring EDS using two single questions^21^. Among participants with EDS at baseline, 21% only persisted at the 10-year follow-up^21^. Thus, EDS appears to be an unstable symptom in the general population and cannot be considered as a disorder *per se* ^24^.

In addition to changes in the trajectory of sleepiness propensity with time, we investigated the test-retest reliability of the tool. Despite its widespread use, and its good psychometric properties in students^22^, a systematic review of the ESS showed poor test-retest reliability especially in middle-aged populations and after a time period greater than one year^25^. Furthermore, despite validated norms in healthy subjects with scores between 0 and 10, a grey zone for pathological scores existed with doubt for some conditions due to potential difficulties in understanding the questions and the quality of the subject’s perception of their sleepiness. Moreover, significant changes within the scores may occur even under the threshold (i.e. the “presymptomatic stage”), or already above the threshold referring to an aggravation of the condition. Accordingly, we reanalyzed the ESS scores when there was an increase of 4-points or more between two consecutive evaluations. As already reported in the literature^17, 18^ such an important difference in DS may be considered as clinically meaningful and should therefore alert the clinician. Results showed that 33% had increased DS but only 36% were persistent; although this is low, it is twice as high as for persistent EDS.

Other important findings of our study concerned the risk factors associated with incident EDS and DS increase. We found that younger age was an independent predictor of incident EDS and DS increase but did not observe a U-shaped relationship as previously described for EDS^21^. We replicated the association between depressive symptoms and incident EDS and DS increase shown in others studies^20, 21^. We also reported that lifestyle factors (*i*.*e*. lower coffee consumption and smoking) were associated with incident EDS and living alone with DS increase only, all factors being modifiable on a behavioral level. Smoking as previously reported associated with the development of EDS^20^. These two lifestyle factors were related to EDS with mechanisms of action being potentially related to the stimulant effect of caffeine and the non-restorative sleep effect of nicotine^26^. In the same way, insomnia, fatigue and chronic pain, being markers of poor health status, were associated with incident EDS. Fatigue and insomnia were already reported as potential risk factors for EDS but to our knowledge we reported for the first time that chronic pain may be a risk factor for EDS in the general population. The mechanism underlying this association remains unknown but it may involve sleep fragmentation and chronic low-grade inflammation^27, 28^. Several arguments already suggest a complex relationship between insomnia, hypersomnia and chronic pain^29^. We failed to report any relationship between change of BMI classes and EDS/DS onset. This discrepancy may relate to the low proportions of subjects with obesity (~20%) and with significant BMI changes in our sample (~15%). Last, typical short sleep duration was associated with incidence of EDS and DS increase in bivariate models with similar tendency for the former outcome in multivariate model. Finally, potential association with long sleep duration defined as >9 hours in the literature^30^ could not be assessed here as it corresponds to only 6% (n = 88) of the population.

We also identify predictors of persistent *vs* transient EDS or DS increase that showed associations with poor general health including somatic diseases and especially metabolic diseases. None of lifestyle and psychological factors were associated with persistent sleepiness. Findings focusing on subjects with persistent sleepiness are of major interest as they highlight a more homogeneous group potentially at risk for serious medical consequences such as cardiovascular and neurodegenerative disorders. Interventional studies exploring whether the management of sleep complaint improves or prevents the medical condition, and whether the management of the medical condition improves sleep symptoms, are warranted in these subjects with persistent sleepiness.

The strengths of this present study are the prospective design, the size of the cohort and the large number of potential risk factors including socio-demographic, lifestyle, health and psychological variables. The study had also a long follow-up of five years with yearly evaluations allowing assessment of potential fluctuation of sleepiness course and changes in covariates over time. Furthermore, the evaluation of EDS was assessed using the ESS which is the most commonly used tool in sleep research and clinical setting.

This study has some limitations. Selection bias may exist even if random selection was used for the recruitment of the participants. The data are self-reported with possible recall and common method bias. The responses when filling the questionnaire may depend on the participant’s perceptual experience. Objective measures of sleep quality and DS by polysomnographic (PSG) and multiple sleep latency test (MSLT) recordings were not available for this study; however, performing PSG and MSLT were very difficult within the context of a large epidemiological survey. Moreover the agreement between objective and subjective EDS was always unsatisfactory.

In conclusion, our study reports a high fluctuation of EDS and increased DS over time in the general population. Sleepiness is predicted by a similar set of lifestyle and psychological factors that are potentially modifiable. In contrast, persistent EDS or increased DS are associated with chronic medical diseases thus individualizing a homogeneous group at risk of serious medical consequences requiring a dedicated management.

## Methods

### Study population

Subjects included were recruited as part of a larger epidemiological study aiming to assess prevalence and incidence, risk factors, natural history, and burden of insomnia in Canada. Detailed design and sampling procedures for this study have been reported elsewhere^31^. Briefly, subjects aged ≥18 years were recruited from a random selection of more than 12000 subjects who completed a telephone interview about their sleep between 2007 and 2008. Participants were then asked if they wanted to take part in the longitudinal phase of the study, which involved completion of seven postal evaluations over a 5-year period: the baseline evaluation was sent one month after the telephone interview, the second at 6 months and the remaining evaluations scheduled every years. Overall, 3006 completed baseline postal assessment. All participants provided written informed consent to participate in the study, prior to the study protocol and approved by the ethical committee of the Université Laval-Quebec, Canada. The methods in the current study were carried out in accordance with the approved guidelines.

### EDS and DS increase during the 5-year follow-up

EDS was assessed at baseline and at each follow-up wave (after 6 months, 1, 2, 3, 4 and 5 years) by the ESS^14^. This scale comprises eight items evaluating sleepiness on a Likert scale from 0 to 3; total scores vary between 0 and 24. A total score >10 indicates EDS, and 15 severe EDS.

New cases of EDS between two consecutive visits were defined as participants with EDS at year ‘t’ but without EDS at year ‘t-1’. Remitted EDS cases were defined as participants without EDS at year ‘t’ but with EDS at year ‘t-1’, and recurrent EDS cases as participants with EDS at year ‘t-1’ and at year ‘t’.

In longitudinal analyses, the incidence of EDS was identified from participants without EDS at baseline but who subsequently developed EDS in at least one the follow-up evaluations. Persistent EDS cases were defined as participants reporting EDS at “t”-year follow-up that persists until the end of follow-up. Transient EDS cases was defined as participants reporting EDS at “t”-year follow-up without persistence until the end of follow-up.

A significant increase of DS during the follow-up was defined by at least a 4-point augmentation on the ESS scale between two consecutive evaluations that can or not cross the cut-off of 10. A 4-point change was considered clinically-relevant in previous pharmacological research studies on hypersomnia^17, 18^ and statistically, it corresponds to the lowest decile of all differences between two consecutive ESS scores. However, an augmentation of 4 points from previous ESS score equal to 0, 1, 2 and 3 was ruled out as considered non-clinically significant (N = 42). Persistent DS increase was defined as the absence of ESS decrease of more than 3-point in the subsequent follow-ups.

### Other sleep-related measures

At each evaluation, sleep duration during the last month was self-reported by participant and divided into 3 categories: <6.0 hours/night, between 6.0 and 7.9, ≥8 based on published literature^10^. The number of naps during the last month was recorded as none; <1 per week; 1 or 2; ≥3. The severity of insomnia was evaluated using the Insomnia Severity Index (ISI)^32^. This seven-item questionnaire assesses the nature, severity, and impact of sleep difficulties yielding a total score ranging from 0 to 28. The total score is interpreted as follows: absence of insomnia (0–7); sub-threshold insomnia (8–14); moderate insomnia (15–21); and severe insomnia (22–28).

A standardized questionnaire was completed relative to diagnosis made by healthcare professionals on sleep disorders including central hypersomnias, insomnia, sleep apnea syndrome, and restless legs syndrome.

Sleep medication including prescriptions and over-the-counter (OTC) drugs used in the previous month were systematically detailed and classified as benzodiazepine (BZD), BZD-like compounds (zolpidem, zopiclone, zaleplon), antidepressants and miscellaneous medications (including barbiturates, OTC, and neuroleptics).

### Socio-demographic, lifestyle, health and psychological measures

Standardized evaluation at baseline and at each follow-up included questions related to demographic characteristics, level of education (high school/college; university level), marital status (categorized as being single, divorced/separated or widowed; married, common-law couple), daily life habits such as alcohol consumption (categorized as none; 1 drink per day; ≥2 drinks), caffeine intake (categorized as none; 1–2 cups per day; ≥3), smoking status (classified as never; present or past users), and physical activity (categorized as never or less than one per week; between 1 and 3 per week, more than 4 times per week) and anthropometric data with height and weight to calculate body mass index (BMI) (classified as <25 kg/m^2^: normal; 25–30: overweight; ≥30: obese).

History of diabetes, endocrine and metabolic disorders (e.g. cholesterol, dysthyroidism), hypertension, cardio-vascular diseases, chronic pain, neurological diseases (epilepsy, headache, migraine) and other diseases including allergies, cancer, digestive, bone, lung, otorhinolaryngology, skin, urinary or genital problems was established at baseline and at each follow-up using standardized questions.

Standardized questionnaires were also completed at baseline and at each follow-up. Depressive symptoms were evaluated using the Beck Depression Inventory II (BDI-II)^33^. This scale contains 21 questions, with a total score ranging from 0 to 63, with higher scores suggesting more severe depressive symptoms (≤13: minimal; 14–19: mild, 20–28: moderate and 29–69: severe depression). State-Trait Anxiety Inventory (STAI) was used to measure state and trait anxiety symptoms^34^. The two subscales are composed of 20 items with overall scores ranging from 20 to 80. In the absence of validated cut-off score, STAI trait and state scores were divided into tertiles. General fatigue was assessed by the multidimensional fatigue inventory (MFI)^35^. The dimension of General Fatigue was used with a score varying between 4 and 20. The score was also divided into tertiles.

### Statistical analysis

Associations between the characteristics of participants and EDS (ESS > 10 *vs*. ≤10) at baseline were quantified with odds ratios (OR) and their 95% confidence intervals (CI). Age, gender and other sociodemographic, health behavior and psychological variables associated with EDS at p < 0.15 in bivariate analysis were entered in a logistic regression model to determine which characteristics were independently associated with the presence of EDS. To study which factors were independently associated with EDS severity, three groups of subjects with ESS score ≤10, between 11 and 15 and ≥16 were compared by multinomial logistic regression model with age, gender and factors associated with the severity at p < 0.15. The p-value cut-off choice of 0.15 was based on the recommendations of several authors to use a significance level as high as 0.15, for variable selection in the bivariate analysis because such as 0.05 can fail to identify variables known to be important^36^.

Cox proportional hazard models were used to estimate hazard ratios (HR) and their 95% CI for the associations between potential predictors of a first event of EDS in bivariate analysis. Covariables that appeared or changed during the follow-up were taken into account as time-dependent covariates in the Cox proportional hazard models. The covariate values the year prior to the event time was taken but if this covariate was not updated at an annual visit, then the prior value was carried forward in the time-dependent model. In the case of multiple events during the follow-up, the first event was considered in the analysis. The multivariate analysis, which included age, gender and variables associated at p < 0.15 in the bivariate analysis, was performed using Cox proportional hazard model to evaluate which predictors were independently associated with the incidence of EDS. Similar methodology was performed for the study of predictors of incident DS during the 5-year follow-up. Significance level was set at p < 0.05. Analyses were performed using SAS-version 9.4 (SAS Inc, Cary, NC, USA).

## References

[1] American Academy of Sleep Medicine. *International Classification of Sleep Disorders*, *3rd ed*. (American Academy of Sleep Medicine, 2014).

[2] Akbaraly TN (2015). Sleep complaints and metabolic syndrome in an elderly population: the Three-City Study. Am J Geriatr Psychiatry.

[3] Bixler EO (2005). Excessive daytime sleepiness in a general population sample: the role of sleep apnea, age, obesity, diabetes, and depression. J Clin Endocrinol Metab.

[4] Ohayon MM, Vecchierini MF (2002). Daytime sleepiness and cognitive impairment in the elderly population. Arch Intern Med.

[5] Tsuno N (2007). Determinants of excessive daytime sleepiness in a French community-dwelling elderly population. J Sleep Res.

[6] Empana JP (2009). Excessive daytime sleepiness is an independent risk indicator for cardiovascular mortality in community-dwelling elderly: the three city study. Stroke.

[7] Jaussent I (2011). Insomnia and daytime sleepiness are risk factors for depressive symptoms in the elderly. Sleep.

[8] Joo S (2009). Prevalence of excessive daytime sleepiness and associated factors in the adult population of Korea. Sleep Med.

[9] Klink M, Quan SF (1987). Prevalence of reported sleep disturbances in a general adult population and their relationship to obstructive airways diseases. Chest.

[10] Ohayon MM, Dauvilliers Y, Reynolds CF (2012). Operational definitions and algorithms for excessive sleepiness in the general population: implications for DSM-5 nosology. Arch Gen Psychiatry.

[11] Pallesen S (2007). Prevalence and risk factors of subjective sleepiness in the general adult population. Sleep.

[12] Ohayon MM, Priest RG, Zulley J, Smirne S, Paiva T (2002). Prevalence of narcolepsy symptomatology and diagnosis in the European general population. Neurology.

[13] Ohayon MM (2008). From wakefulness to excessive sleepiness: what we know and still need to know. Sleep Med Rev.

[14] Johns MW (1991). A new method for measuring daytime sleepiness: the Epworth sleepiness scale. Sleep.

[15] Souza JC, Magna LA, Reimao R (2002). Excessive daytime sleepiness in Campo Grande general population, Brazil. Arq Neuropsiquiatr.

[16] Wu S (2012). Excessive daytime sleepiness assessed by the Epworth Sleepiness Scale and its association with health related quality of life: a population-based study in China. BMC public health.

[17] Dauvilliers Y (2013). Pitolisant versus placebo or modafinil in patients with narcolepsy: a double-blind, randomised trial. The Lancet. Neurology.

[18] US Modafinil in Narcolepsy Multicenter Study Group (2000). Randomized trial of modafinil as a treatment for the excessive daytime somnolence of narcolepsy: US Modafinil in Narcolepsy Multicenter Study Group. Neurology.

[19] Hasler G (2005). Excessive daytime sleepiness in young adults: a 20-year prospective community study. J Clin Psychiatry.

[20] Theorell-Haglow J, Akerstedt T, Schwarz J, Lindberg E (2015). Predictors for Development of Excessive Daytime Sleepiness in Women: A Population-Based 10-Year Follow-Up. Sleep.

[21] Fernandez-Mendoza J (2015). Natural history of excessive daytime sleepiness: role of obesity, weight loss, depression, and sleep propensity. Sleep.

[22] Johns MW (1992). Reliability and factor analysis of the Epworth Sleepiness Scale. Sleep.

[23] Kaminska M (2010). The Epworth Sleepiness Scale: self-administration versus administration by the physician, and validation of a French version. Can Respir J.

[24] Dauvilliers Y, Lopez R, Ohayon M, Bayard S (2013). Hypersomnia and depressive symptoms: methodological and clinical aspects. BMC Med.

[25] Kendzerska TB, Smith PM, Brignardello-Petersen R, Leung RS, Tomlinson GA (2014). Evaluation of the measurement properties of the Epworth sleepiness scale: a systematic review. Sleep Med Rev.

[26] Soldatos CR, Kales JD, Scharf MB, Bixler EO, Kales A (1980). Cigarette smoking associated with sleep difficulty. Science.

[27] Irwin MR, Olmstead R, Carroll JE (2016). Sleep Disturbance, Sleep Duration, and Inflammation: A Systematic Review and Meta-Analysis of Cohort Studies and Experimental Sleep Deprivation. Biol Psychiatry.

[28] Vgontzas AN, Bixler EO, Chrousos GP (2006). Obesity-related sleepiness and fatigue: the role of the stress system and cytokines. Annals of the New York Academy of Sciences.

[29] Roehrs T, Roth T (2005). Sleep and pain: interaction of two vital functions. Seminars in neurology.

[30] Ohayon MM, Reynolds CF, Dauvilliers Y (2013). Excessive sleep duration and quality of life. Ann Neurol.

[31] Morin CM (2009). The natural history of insomnia: a population-based 3-year longitudinal study. Arch Intern Med.

[32] Bastien CH, Vallieres A, Morin CM (2001). Validation of the Insomnia Severity Index as an outcome measure for insomnia research. Sleep Med.

[33] Beck, A. T., Steer, R. A. & Brown, G. K. *Inventaire de dépression de Beck- deuxième édition* Toronto: Pyschological Corporation edn (1996).

[34] Spielberger, C. *Manual for the State-Trait anxiety Inventory* (*Form Y*) (Palo Alto, CA: Consulting Psychologists Press, 1983).

[35] Smets EM, Garssen B, Bonke B, De Haes JC (1995). The Multidimensional Fatigue Inventory (MFI) psychometric qualities of an instrument to assess fatigue. J Psychosom Res.

[36] Hosmer, D. & Lemeshow, S. *Applied Logistic Regression* Second Edition (Wiley inter-science, 2000).

